# A Single Mutation in Chikungunya Virus Affects Vector Specificity and Epidemic Potential

**DOI:** 10.1371/journal.ppat.0030201

**Published:** 2007-12-07

**Authors:** Konstantin A Tsetsarkin, Dana L Vanlandingham, Charles E McGee, Stephen Higgs

**Affiliations:** Department of Pathology, University of Texas Medical Branch, Galveston, Texas, United States of America; The Pennsylvania State University, United States of America

## Abstract

Chikungunya virus (CHIKV) is an emerging arbovirus associated with several recent large-scale epidemics. The 2005–2006 epidemic on Reunion island that resulted in approximately 266,000 human cases was associated with a strain of CHIKV with a mutation in the envelope protein gene (E1-A226V). To test the hypothesis that this mutation in the epidemic CHIKV (strain LR2006 OPY1) might influence fitness for different vector species, viral infectivity, dissemination, and transmission of CHIKV were compared in *Aedes albopictus*, the species implicated in the epidemic, and the recognized vector *Ae. aegypti.* Using viral infectious clones of the Reunion strain and a West African strain of CHIKV, into which either the E1–226 A or V mutation was engineered, we demonstrated that the E1-A226V mutation was directly responsible for a significant increase in CHIKV infectivity for *Ae. albopictus*, and led to more efficient viral dissemination into mosquito secondary organs and transmission to suckling mice. This mutation caused a marginal decrease in CHIKV *Ae. aegypti* midgut infectivity, had no effect on viral dissemination, and was associated with a slight increase in transmission by *Ae. aegypti* to suckling mice in competition experiments. The effect of the E1-A226V mutation on cholesterol dependence of CHIKV was also analyzed, revealing an association between cholesterol dependence and increased fitness of CHIKV in *Ae. albopictus*. Our observation that a single amino acid substitution can influence vector specificity provides a plausible explanation of how this mutant virus caused an epidemic in a region lacking the typical vector. This has important implications with respect to how viruses may establish a transmission cycle when introduced into a new area. Due to the widespread distribution of *Ae. albopictus*, this mutation increases the potential for CHIKV to permanently extend its range into Europe and the Americas.

## Author Summary

Chikungunya virus (CHIKV) is an emerging arbovirus associated with several recent large-scale epidemics of arthritic disease, including one on Reunion island, where there were approximately 266,000 cases (34% of the total island population). CHIKV is transmitted by *Aedes* species mosquitoes, primarily *Ae. aegypti*. However, the 2005–2006 CHIKV epidemic on Reunion island was unusual because the vector responsible for transmission between humans was apparently the Asian tiger mosquito, *Ae. albopictus*. Interestingly, the same epidemic was associated with a strain of CHIKV with a mutation in the envelope protein gene (E1-A226V). In this work we investigated the role of the E1-A226V mutation on the fitness of CHIKV in *Ae. aegypti* and *Ae. albopictus* mosquitoes. We found that E1-A226V is directly responsible for CHIKV adaptation to *Ae. albopictus* mosquitoes, which provides a plausible explanation of how this mutant virus caused an epidemic in a region lacking the typical vector. This research gives a new insight into how a simple genetic change in a human pathogen can increase its host range and therefore its geographic distribution. *Ae. albopictus* is abundant and widely distributed in urban areas of Europe and the United States of America, and this work suggests that these areas are now vulnerable to CHIKV establishment.

## Introduction

The large-scale epidemic of the mosquito-transmitted alphavirus, Chikungunya virus (CHIKV), began in Kenya in 2004 and spread to several Indian Ocean islands including the Comoros, Mauritius, the Seychelles, Madagascar, Mayotte and Reunion. On Reunion island alone there were approximately 266,000 cases (34% of the total island population) [1–6]. In the continuing Indian epidemic there have been at least 1.4M cases reported [7–10] with continued expansion in Sri Lanka and Indonesia. CHIKV had not been reported to cause fatalities in prior outbreaks; however, during the outbreak on Reunion island, CHIKV was associated with at least 260 deaths [11,12]. The strain of CHIKV responsible for the Indian Ocean island epidemic has been well-characterized in cell culture and mosquito models [13–15]; however, the underlying genetic basis of the atypical phenotype of this CHIKV strain remains unknown.

CHIKV is transmitted by *Aedes* species mosquitoes, primarily *Ae. aegypti*. However, the 2005–2006 CHIKV epidemic on Reunion island was unusual because the vector responsible for transmission between humans was apparently the Asian tiger mosquito, *Ae. albopictus* [3,16]. This conclusion is based on several factors. This species is known to be susceptible to CHIKV infection and although infectious virus was not isolated from *Ae. albopictus* during the epidemic, CHIKV RNA was detected (X. de Lamballerie, personal communication). Furthermore, the species is anthropophylic, was abundant during the epidemic, and other potential vectors specifically *Ae. aegypti* were relatively scarce with a very limited distribution (P. Reiter, personal communication). *Ae. albopictus* is abundant and widely distributed in urban areas of Europe and the United States of America [17–22]. CHIKV infections have been reported in many travelers returning to the US and Europe [12,23–26] causing concern that the virus could be introduced and become established in these areas [1,27,28]. In August and September of 2007, a CHIKV–*Ae. albopictus* transmission cycle was reported for the first time in Europe, with an estimated 254 human cases occurring in Italy [29,30].

Alphaviruses are enveloped single stranded positive sense RNA viruses. Genomic RNA, of ≈ 12,000 nt, encodes four non-structural (ns1–4) and three main structural proteins (capsid, E2 and E1). At neutral pH, E2 and E1 exist as heterodimers in which E2 forms spikes on the virion surface that interact with cellular receptors. The E1 protein lies below E2 and mediates fusion of the viral and cellular membranes during viral entry [31].

Analysis of CHIKV genome microevolution during the 2005–2006 Indian Ocean epidemic identified an alanine to valine mutation at position 226 in the E1 envelope glycoprotein (E1-A226V) among viral isolates obtained during the outbreak [32]. The reason for this was unclear but it was hypothesized that the E1-A226V mutation might influence infectivity of CHIKV for mosquito vectors [11,32]. Interestingly, earlier studies have identified that a P→S mutation in the same position of the E1 glycoprotein is responsible for the modulation of Semliki Forest virus's (SFV, a member of the alphavirus family) requirements for cholesterol in the target membrane [33]. It also has been shown that the presence of this mutation results in more efficient growth of SFV in *Ae. albopictus* mosquitoes [34]. However, no evidence has been presented to directly correlate the release from the cholesterol dependence, associated with the E1-P226S mutation in SFV, with a growth advantage in *Ae. albopictus*. It is unknown if dependence on cholesterol for growth in mosquito cells is a requirement of all alphaviruses.

To test the hypothesis that the E1-A226V mutation might influence the fitness of CHIKV in mosquito vectors, we compared the effect of this mutation on CHIKV mosquito infectivity, the ability to disseminate into heads and salivary glands, and the relative fitness in competition assays for transmission by *Ae. albopictus* and *Ae. aegypti* to suckling mice. We also analyzed the effect of the E1-A226V mutation on CHIKV cholesterol dependence for growth in mosquito C6/36 (*Ae. albopictus*) cells. Here we report findings that a single nucleotide change, which arose during the epidemic, significantly increases fitness of the virus for *Ae. albopictus* mosquitoes and was associated with CHIKV dependence on cholesterol in the mosquito cell membrane. This change likely enhanced CHIKV transmission by an atypical vector and contributed to the maintenance and scale of the epidemic.

## Results

### Effect of E1 A226V Mutation on Fitness of CHIKV in *Ae. albopictus* Mosquitoes

To test the hypothesis that the E1-A226V mutation altered CHIKV infectivity for *Ae. albopictus* mosquitoes, CHIKV infectious clones derived from an epidemic Reunion island human isolate were used [15], including one clone (LR-GFP-226V) expressing enhanced green fluorescent protein (eGFP). Clones were further engineered to express E1 protein containing an alanine at position E1–226 (LR-GFP-226A) representing the CHIKV genotype prevalent prior to the outbreak gaining momentum (Figure S1). RNAs produced from both clones (LR-GFP-226V and LR-GFP-226A) have comparable specific infectivity values, produced similar viral titers following transfection into BHK-21 cells (Table S1) and have similar growth kinetics in mosquito (C6/36) and mammalian (BHK-21) cells lines (Figure S2A and S2B).

**Figure 1:**
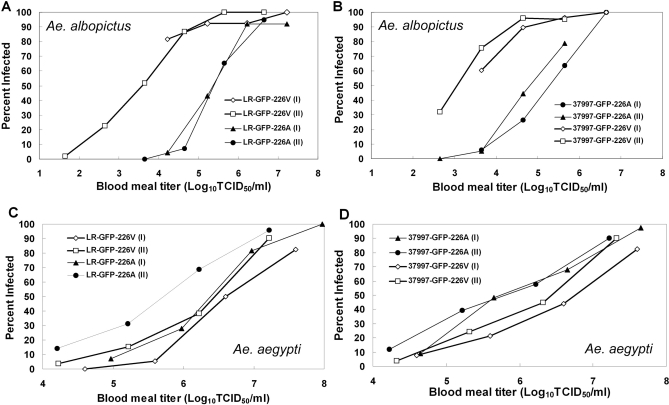
Effect of E1-A226V Mutation on CHIKV-GFP Viruses *Ae. albopictus* and *Ae. aegypti* Midgut Infectivity Percent of orally infected *Ae. albopictus* (A, B) and *Ae. aegypti* (C, D) mosquitoes presented with blood meals containing various concentration of eGFP-expressing CHIK viruses. Serial 10-fold dilutions of viruses in the backbone of Reunion (LR-GFP-226V and LR-GFP-226A) (A, C) and 37997 (37997-GFP-226A and 37997-GFP-226V) (B, D) strains of CHIKV were made in L-15 medium followed by mixing the samples with defibrinated sheep blood. Mosquitoes were dissected at 7 dpi and eGFP expression in infected midguts was analyzed by fluorescence microscopy. A mosquito was considered infected if at least one foci of eGFP-expressing cells was present in the midgut. The experiments were performed twice for each virus (I and II).

The relative infectivity of LR-GFP-226V and LR-GFP-226A viruses was analyzed in female *Ae. albopictus* mosquitoes orally exposed to serial 10-fold dilutions of CHIKV (LR-GFP-226 V or A). To determine whether infection rates correlate with blood meal titer, midguts dissected from mosquitoes at 7 days post-infection (dpi) were analyzed for foci of eGFP-expressing cells by fluorescence microscopy (Figure 1A; Table 1). In two independent experiments, LR-GFP-226V virus was found to be approximately 100-fold more infectious to *Ae. albopictus* than LR-GFP-226A virus (p<0.01). To test if the infectivity phenotype was directly linked to the mutation, the complementary reverse mutation, E1-A226V, was introduced into an infectious clone of a West African CHIKV strain, 37997-GFP (37997-GFP-226A) (Figure S1). The Reunion and 37997 strains of CHIKV are distantly related, with only 85% nucleotide sequence identity. The parental 37997-GFP-226A and the 37997-GFP-226V viruses were indistinguishable in cell culture experiments (Table S1; Figure S2C and S2D); however, *in vivo* experiments in *Ae. albopictus* mosquitoes revealed that the E1-A226V mutation significantly decreases the oral infectious dose 50 (OID_50_) value for the 37997-GFP-226V virus (p<0.01) to an extent similar to that observed for LR-GFP-226V virus (Figure 1B; Table 1). These data conclusively demonstrate that the single E1-A226V point mutation is therefore sufficient to significantly reduce the OID_50_ of the 37997-GFP virus (p<0.01) in *Ae. albopictus* mosquitoes equivalent to that observed for the LR-GFP-226V virus (Figure 1A; Table 1).

**Table 1:**
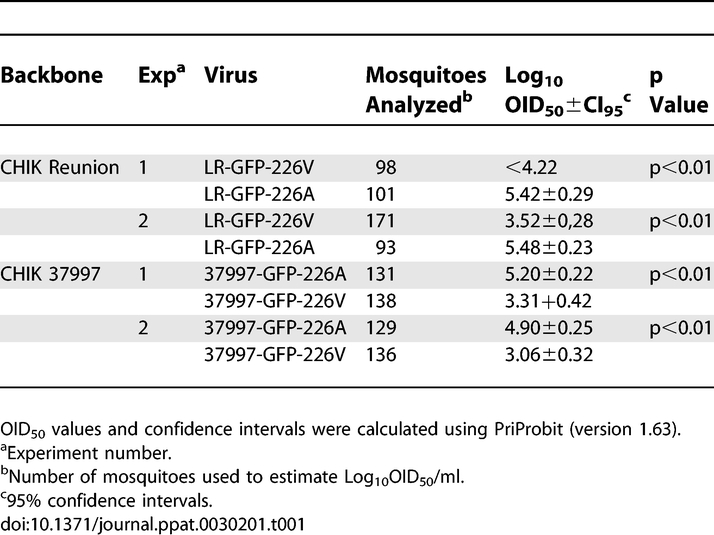
Log_10_OID_50_/ml for CHIKV in *Ae. albopictus* Mosquitoes

**Figure 2:**
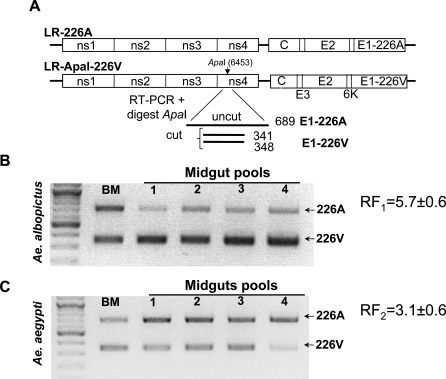
Schematic Representation of Competition Experiments (A) and Competition between LR-ApaI-226V and LR-226A Viruses for Colonization of Midgut cells of *Ae. albopictus* (B) and *Ae. aegypti* (C) Mosquitoes 10^7^ pfu of LR-ApaI-226V and LR-226A were mixed and orally presented to *Ae. albopictus* (B) and *Ae. aegypti* (C). Viral RNAs were extracted from four pools of eight to ten midguts at 7 dpi. RT-PCR products were digested with *Apa*I, separated in 2% agarose gel, and gels were stained using ethidium bromide. BM - initial ratio of LR-ApaI-226V and LR-226A in blood meal samples. 1–4 ratio of LR-ApaI-226V and LR-226A RNA in four independent replicas of the eight to ten midguts per replica. Relative fitness (RF_1_) of LR-Apa-226V to LR-226A was calculated as a ratio between 226V and 226A bands in the sample, divided by the control ratio between 226V and 226A in the blood meal. Relative fitness (RF_2_) of LR-226A to LR-Apa-226V was calculated as a ratio between 226A and 226V bands in the sample, divided to the control ratio between 226A and 226V in the blood meal. Results expressed as the average of four replicas ± standard deviation (SD).

To further evaluate viral fitness of the epidemic CHIKV E1-A226V mutation in *Ae. albopictus*, viral competition experiments were performed. Although our CHIKV eGFP-expressing infectious clones, have similar infection properties in mosquitoes as wild-type viruses [15,35], to address potential concerns that eGFP expression might influence OID_50_ values, we constructed LR-226A and LR-ApaI-226V viruses without eGFP and employed them in viral competition experiments (Figures 2A and S1). LR-ApaI-226V was derived from previously described CHIK-LR ic, by the introduction of a silent marker mutation, A6454C, in order to add an *Apa*I restriction site into the coding sequence. It was shown that the A6454C mutation does not affect the specific infectivity value (Table S1), the viral titer after RNA transfection into BHK-21 cells value (Table S1), the viral growth kinetics in BHK-21 and C6/36 cells (Figure S3), infectivity for and viral titers in *Ae. aegypti* and *Ae. albopictus* mosquitoes (Table S2), or viral fitness for growth in BHK-21 and C6/36 cells as determined by competition assay (Figure S4). These data indicate that the introduced mutation is indeed silent and does not affect the fitness of LR-ApaI-226V.

For viral competition experiments LR-ApaI-226V virus (10^7^ plaque-forming units (pfu)) was mixed with an equal amount of LR-226A virus. LR-ApaI-226V and LR-226A viruses are indistinguishable in cell culture experiments (Figure S3). Mixtures of LR-ApaI-226V and LR-226A viruses were orally presented to *Ae. albopictus* mosquitoes in a blood meal, and midguts were examined at 7 dpi. The relative amount of RNA derived from LR-ApaI-226V in the midgut cells increased 5.7±0.6 times as compared to the initial relative amount of LR-ApaI-226V RNA in the blood meal sample (Figure 2B). These data support our observation that the E1-A226V mutation enhances infectivity of CHIKV for *Ae. albopictus* mosquitoes and furthermore demonstrate that the mutation could provide an evolutionary advantage over E1-226A viruses in an atypical vector and may have perpetuated the outbreak in a region where *Ae. albopictus* was the predominant anthropophilic mosquito species.

**Figure 3:**
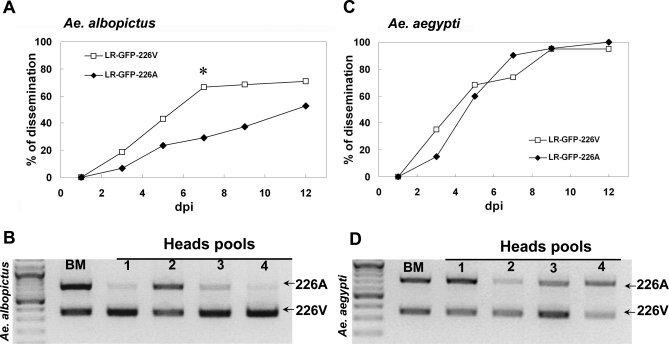
Effect of E1-A226V Mutation on CHIKV Dissemination into Salivary Glands and Heads of *Ae. albopictus* and *Ae. aegypti* Mosquitoes *Ae. albopictus* (A) and *Ae. aegypti* (C) mosquitoes were orally infected with LR-GFP-226V and LR-GFP-226A. At the indicated time points, 16–21 mosquitoes were dissected and salivary glands were analyzed for eGFP expression. Percent of dissemination was estimated as a ratio of the number of mosquitoes with eGFP-positive salivary glands to the number of mosquitoes with eGFP-positive midguts. For *Ae. albopictus*, infectious blood meal titers were 5.95 and 6.52 Log_10_TCID_50_/ml for LR-GFP-226V and LR-GFP-226A, respectively. For *Ae. aegypti*, the infectious blood meal titer was 6.95 Log_10_TCID_50_/ml for both LR-GFP-226V and LR-GFP-226A viruses. Dissemination rates were compared statistically by Fisher's exact test using SPSS version 11.5. Asterisk indicates p < 0.05. (B and D) Competition between LR-ApaI-226V and LR-226A for dissemination into heads of *Ae. albopictus* and *Ae. aegypti* mosquitoes. 10^7^ pfu of LR-ApaI-226V and LR-226A were mixed and orally presented to *Ae. albopictus* (B) and *Ae. aegypti* (D). Viral RNAs were extracted from four pools of five heads collected at 12 dpi. RT-PCR products were digested with *Apa*I, separated in 2% agarose gel, and gels were stained using ethidium bromide. BM - initial ratio of LR-ApaI-226V and LR-226A in blood meal samples. 1–4 ratio of LR-ApaI-226V and LR-226A RNA in four independent replicas of the five pooled heads per replica.

**Figure 4:**
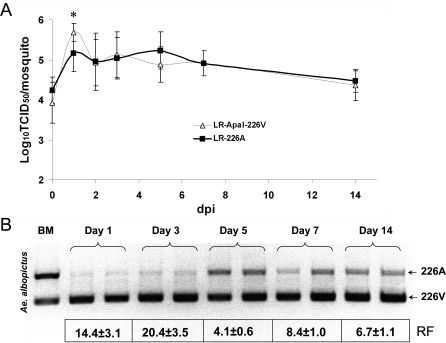
Effect of E1-A226V Mutation on CHIKV Kinetics of Viral Growth in Bodies of *Ae. albopictus* Mosquitoes (A) Virus production in orally infected *Ae. albopictus* mosquitoes. Infected mosquitoes were sampled at 0, 1, 2, 3, 5, 7, and 14 dpi and titrated on Vero cells to estimate average titer ± standard deviation of eight whole mosquitoes. Differences in viral titers were analyzed by pairwise t-tests. Asterisk indicates p < 0.05. (B) Kinetics of competition between LR-ApaI-226V and LR-226A in bodies of *Ae. albopictus* mosquitoes. 10^7^ pfu of LR-ApaI-226V and LR-226A were mixed and orally presented to *Ae. albopictus.* Infected mosquitoes were sampled at 1, 3, 5, 7, and 14 dpi. For each time point, viral RNA was extracted from two pools of ten mosquitoes. BM - initial ratio of LR-ApaI-226V and LR-226A in blood meal samples. RF - relative fitness of LR-Apa-226V to LR-226A was calculated as a ratio between 226V and 226A bands in the sample, divided to the control ratio between 226V and 226A in the blood meal. Results expressed as average of two replicas ± standard deviation.

To determine if the enhanced midgut infectivity associated with the E1-A226V mutation may result in more efficient viral dissemination into secondary tissues, the kinetics of viral dissemination by LR-GFP-226V and LR-GFP-226A into salivary glands, and competition between LR-ApaI-226V and LR-226A for dissemination into mosquito heads were analyzed (Figure 3A and 3B). LR-GFP-226V virus disseminated more rapidly into *Ae. albopictus* salivary glands at all time points, with a significant difference at 7 dpi (p=0.044, Fisher's exact test). Similarly, in three of four replicates of competition experiments, RNA from LR-ApaI-226V virus was dramatically more abundant in the heads of *Ae. albopictus* mosquitoes as compared to RNA from LR-226A (Figure 3B, lines 1, 3, 4), although in one replica LR-ApaI-226V RNA was only slightly more abundant as compared to the initial viral RNA ratio (Figure 3B, line 2). This variability of the results may be due to random pooling of mosquito heads. Thus, replicate two may have included more heads negative for LR-Apal-226V relative to heads positive for LR-226A RNA. Another possibility is that at some point during viral dissemination from the midguts into mosquito heads, LR-226A may replicate more rapidly than LR-ApaI-226V. To further investigate this relationship, *Ae. albopictus* mosquitoes were orally presented with either LR-ApaI-226V or LR-226A and whole mosquito body viral titers were compared at different time points pi. Surprisingly, no significant differences between viral titers were found, with the exception of 1 dpi, where the LR-ApaI-226V titer was 0.5 Log_10_ tissues culture infectious dose 50 percent end point titer (Log_10_ TCID_50_/mosquito) higher than of the LR-226A titer (Figure 4A). This may be due to more efficient colonization of *Ae. albopictus* midguts by LR-ApaI-226V. The absence of significant differences in viral titers at later time points may be due to variation in viral titers among individual mosquitoes. Competition between LR-ApaI-226V and LR-226A was analyzed at different time points in order to investigate the relationship between replication of LR-ApaI-226V and LR-226A viruses in *Ae. albopictus* mosquitoes (Figure 4B). As expected, the viral RNA from LR-ApaI-226V was predominant at the early time points of 1 and 3 dpi. Interestingly, between 3 and 5 dpi the viral RNA ratio shifted toward LR-226A virus indicating that at these time points, LR-226A replicates more efficiently in some mosquito tissues (Figure 4B). This short period of time may have a slight effect on the overall outcome of competition for dissemination into salivary glands because there is a reverse shift in the RNA ratio between days 5 and 7 toward LR-ApaI-226V virus, which continues through 14 dpi. These data indicate that the E1-A226V mutation not only increases midgut infectivity but also is associated with more efficient viral dissemination from the midgut into secondary organs, suggesting that the E1-A226V mutation would increase transmissibility of CHIKV by *Ae. albopictus* mosquitoes.

**Figure 5:**
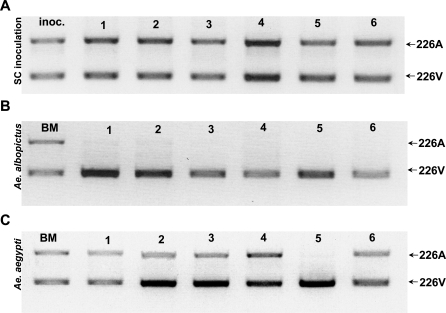
Effect of E1-A226V Mutation on CHIKV Transmission by *Ae. albopictus* and *Ae. aegypti* Mosquitoes (A) Six 2- to 3-day-old suckling mice (Swiss Webster) were subcutaneously infected with a 20-μl mixture of ≈ 25 pfu LR-Apa-226V and ≈ 25 pfu of LR-226A viruses. (B and C) *Ae. aegypti* and *Ae. albopictus* mosquitoes were presented with a blood meal containing 10^7^ pfu/ml of LR-Apa-226V and 10^7^ pfu/ml of LR-226A viruses. At 13 dpi, ten to 15 mosquitoes were placed in separate paper cartons and starved for 24 h. The next day, the mosquitoes in each carton were presented with a 2- to 3-day-old suckling mouse (Swiss Webster). Mice were returned to their cage and sacrificed on day 3 post-exposure. Blood from each individual mouse (≈ 50 μl) was collected and immediately mixed with 450 μl of TRIzol reagent for RNA extraction. BM and inoc. - initial ratio of LR-ApaI-226V and LR-226A in blood meal samples and inoculum for subcutaneous infection. 1–6 ratio of LR-ApaI-226V and LR-226A RNA in six individual mice.

A competition assay between LR-ApaI-226V and LR-226A viruses was used to examine transmission by *Ae. albopictus* to suckling mice to assess the potential for the E1-A226V mutation to influence virus transmission. *Ae. albopictus* mosquitoes were orally presented with a mixture of LR-ApaI-226V and LR-226A viruses and at 14 dpi were allowed to feed on suckling mice. Mice were sacrificed and bled on day 3 following exposure and the presence of CHIKV RNA in the blood was analyzed by RT-PCR followed by restriction digestion with *Apa*I (Figure 5B). Blood obtained from 100% of experimental mice contained detectible amounts of viral RNA, indicating that virus was transmitted by *Ae. albopictus* mosquitoes to suckling mice. More importantly, in all six mice analyzed, RNA derived from LR-ApaI-226V was the predominant viral RNA species, indicating that under the conditions of competition for transmission, the E1-A226V mutation directly increases CHIKV transmission by *Ae. albopictus* mosquitoes. Interestingly, in the control experiment in which mice were subcutaneously inoculated with ≈ 50 pfu of 1:1 mixture of LR-ApaI-226V and LR-226A viruses, RNAs from both viruses were readily detected and no difference was observed in the viral RNA ratio 3 dpi (Figure 5A) indicating that at least in mice, E1-A226V is not associated with changes in viral fitness.

### Effect of E1 A226V Mutation on Fitness of CHIKV in *Ae. aegypti* Mosquitoes

Since the E1-A226V mutation confers a fitness advantage in *Ae. albopictus*, it is unknown why this mutation had not been observed previously. It is possible that this change might have a deleterious effect on viral fitness in the vertebrate host, although our data of direct competition of LR-ApaI-226V and LR-226A viruses in suckling mice (Figure 5A) and analysis of CHIKV cellular tropism of four clinical isolates from Reunion (which have either A or V at position E1–226) [14], suggest that this is unlikely. An alternative hypothesis is that the E1-A226V mutation might compromise the fitness of CHIKV or have neutral fitness effects in the mosquito species which served as a vector for CHIKV prior to its emergence on Reunion island. Since *Ae. aegypti* has generally been regarded as the main vector for CHIKV prior to the emergence on Reunion island, we analyzed the effect of the E1-A226V mutation on fitness of CHIKV in *Ae. aegypti*.

In contrast to the results obtained in *Ae. albopictus* mosquitoes, OID_50_ values of viruses containing the E1-226V in the backbone of the Reunion and 37997 strains of CHIKV were approximately 0.5 Log_10_OID_50_/ml higher than the OID_50_ values of E1-226A viruses in all experiments using *Ae. aegypti*. These differences were statistically significant for one out of two replicates for each virus pair (Figure 1C and 1D; Table 2). A competition assay examining LR-ApaI-226V and LR-226A virus infection in *Ae. aegypti* midguts, demonstrated that LR-226A virus out-competed LR-ApaI-226V virus at 7 dpi in all four replicates using ten midguts per replicate and that the amount of LR-226A RNA increased on average 3.1 times as compared to the initial blood meal RNA ratio (Figure 2C). These data suggest that the E1-A226V mutation has a slight negative effect on CHIKV infectivity of *Ae. aegypti* midguts.

**Table 2:**
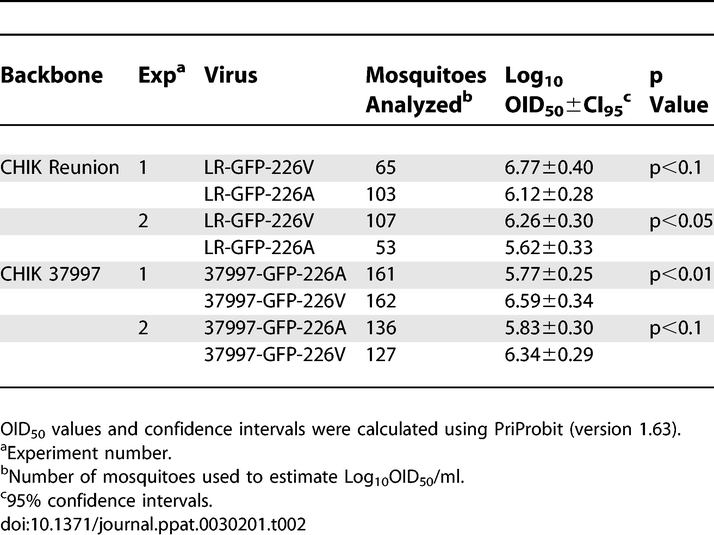
Log_10_OID_50_/ml for CHIKV in *Ae. aegypti* Mosquitoes

The effect of the E1-A226V mutation on the ability of CHIKV to disseminate into *Ae. aegypti* secondary organs was also analyzed (Figure 3C and 3D). LR-GFP-226V and LR-GFP-226A viruses both have similar kinetics of dissemination into salivary glands following oral infection using titers 1–2 Log_10_TCID_50_ higher than their OID_50_ value in *Ae. aegypti* (Figure 3C). In a competition assay, both LR-ApaI-226V and LR-226A viruses disseminated similarly into the heads of *Ae. aegypti*. In two of four replicas, there was a slight increase in the relative amount of LR- 226A RNA (Figure 3D, lines 1, 4); whereas the other two replicas showed a decrease in LR-226A RNA (Figure 2D, lines 2, 3), relative to the initial ratio of the RNA of LR-ApaI-226V and LR-226A viruses in the blood meal. A competition of LR-ApaI-226V and LR-226A viruses for transmission by *Ae. aegypti* to suckling mice was also analyzed (Figure 5C). In contrast to transmission by *Ae. albopictus* mosquitoes, five out of six mice fed upon by *Ae. aegypti* contained comparable amounts of RNA derived from both viruses and only one out of six mice contained RNA derived exclusively from LR-ApaI-226V.

### E1-A226V Mutation Modulates Cholesterol Dependence of CHIKV

**Figure 6:**
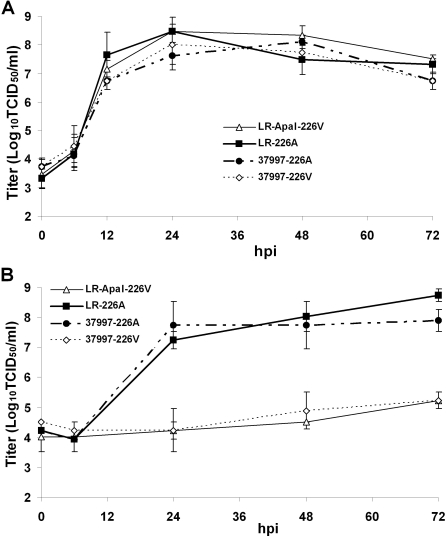
Effect of E1-A226V Mutation on *In Vitro* Growth of CHIKV in Standard (A) and Cholesterol-Depleted (B) C6/36 Cells Cholesterol-depleted C6/36 cells were produced by five passages in L-15 medium containing 10% FBS treated with 2% CAB-O-Sil for 12 h at room temperature as previously described [52]. Confluent monolayers of standard (A) and cholesterol-depleted (B) C6/36 cells were infected with LR-ApaI-226V, LR-226A, 37997–226A and 37997–226V viruses at an MOI of 1.0 (A) and an MOI of 0.1 (B). Cells were washed three times with L-15 medium, and 5.5 ml of fresh L-15 supplied with 10% of standard or CAB-O-Sil-treated FBS were added to the flask. Cells were maintained at 28 °C. At the indicated times post-infection, 0.5 ml of medium was removed and stored at −80 °C for later titration on Vero cells. Viral titers are estimated as average Log_10_TCID_50_/ml ± standard deviation of two independent experiments. hpi - hours post-infection.

It has been previously shown that a P→S mutation in the same E1–226 position of SFV releases cholesterol dependence of the virus in C6/36 cells [33] and results in significantly more rapid growth of SFV in *Ae. albopictus* mosquitoes after intrathoracic inoculation [34]. To determine if a requirement for cholesterol in the cell membrane is important for CHIKV, we analyzed cholesterol dependence of CHIKV E1-226A and E1-226V viruses (Figure 6). Growth curves of E1-226A and E1-226V viruses in the background of Indian Ocean and West African strains of CHIKV were almost indistinguishable when grown in C6/36 cells maintained in L-15 supplied with standard 10% FBS (Figure 6A). However, when the cells were depleted of cholesterol, LR-226A and 37997–226A viruses replicated significantly more rapidly than LR-226V and 37997–226V viruses, reaching 3 Log_10_TCID_50_/ml higher titer at 1, 2 and 3 dpi (Figure 6B). These data indicate that adaptation of CHIKV to *Ae. albopictus* mosquitoes coincides with CHIKV dependence on cholesterol in the target cell membrane.

## Discussion

The CHIKV outbreak in Reunion is unique because it is the first well-documented report of an alphavirus outbreak for which *Ae. albopictus* was the main vector. Interestingly, this was also the first Chikungunya epidemic during which fatal infections were reported. Our data clearly indicate that an E1-A226V mutation in CHIKV results in increased fitness of CHIKV in *Ae. albopictus* mosquitoes with respect to midgut infectivity, dissemination to the salivary glands, and transmission to a vertebrate species. These data demonstrate that a single E1-A226V mutation is sufficient to dramatically increase the ability of different strains of CHIKV to infect *Ae. albopictus* mosquitoes and that this substitution requires no additional adaptive mutations to gain intermolecular compatibility. These complimentary experimental data demonstrate that a single mutation is sufficient to modify viral infectivity for a specific vector species and as a consequence, can fuel an epidemic in a region that lacks the typical vector. These observations provide the basis for an explanation of the observed rapid shift among CHIKV genotypes to viruses containing the E1-A226V mutation during the Reunion outbreak [32].

Interestingly, our data and data from previous studies [36,37] indicate that prior to acquiring the E1-A226V mutation, CHIKV is capable of producing high enough viremia in humans to efficiently infect *Ae. albopictus* mosquitoes. One explanation of the evolutionary force which allowed CHIKV to be selected so rapidly into a CHIKV strain which is adapted to *Ae. albopictus*, is that the increased infectivity (lower OID_50_) of CHIKV E1-A226V mutants for *Ae. albopictus* means that the human viremic thresholds required for *Ae. albopictus* infection would likely occur earlier and be sustained for longer. Several recent studies indicate that during the course of human viremia, which last up to 6 days, CHIKV loads can reach up to 3.3x10^9^ RNA copies per ml of the blood [38,39], which corresponds to 6–7 Log_10_TCID_50_/ml [39]. Earlier studies that utilized a suckling mouse brain titration protocol, which is more sensitive than titration on Vero cells, also found that human viremia often exceeded 6 Log_10_SMICLD_50_/0.02 ml [40]. Based on viremia studies in rhesus monkeys that can develop up to 7.5 Log/ml if assayed by suckling mice brain titration [41] and a maximum viremia of only 5.5 Log_10_/ml based on Vero cell titration [42], we believe that viremias in humans would correlate to 6–7 Log_10_TCID_50_/ml. From these data we calculate that the maximum virus load which can be achieved in human blood is 1–2 Log_10_TCID_50_/ml higher than the Log_10_OID_50_/ml for E1-226A viruses but 3–4 Log_10_TCID_50_/ml higher than the Log_10_OID_50_/ml for E1-226V viruses. During the course of viremia there should therefore be a substantial time frame in which CHIKV blood load is high enough for E1-226V viruses to infect *Ae. albopictus* but below the threshold for infection with E1-226A viruses. This increased opportunity for *Ae. albopictus* infection, would perpetuate the selection and transmission of the mutant virus.

During transmission competition assays, only E1-226V virus was transmitted to suckling mice by *Ae. albopictus*, although in these experiments, titers of E1-226V and E1-226A viruses were of a high enough magnitude to allow both of these viruses to efficiently infect this mosquitoes species. This indicates that there are additional mechanisms that could ensure evolutional success of the E1-A226V viruses transmitted by *Ae. albopictus*. It is possible that one of these mechanisms is associated with more efficient dissemination of the E1-226V as compared with E1-226A viruses. This could shorten the extrinsic incubation period (EIP)—the time from mosquito infection to transmission—and could have contributed to the evolutionary success of CHIKV during the Reunion outbreak because vectors infected with the LR-226V virus would transmit it more quickly than those infected with LR-226A viruses. Additionally, with relatively short-lived vectors such as mosquitoes [43], longer EIPs reduce transmission efficiency simply because fewer mosquitoes survive long enough to transmit the virus.

Our current studies do not provide data to determine if dissemination efficiency of the E1-226V viruses into the salivary glands is a consequence of more efficient midgut infectivity or if these two phenomena are independent. In this regard, it will be of particular interest to investigate the effect of the E1-A226V mutation on CHIKV transmission by orally or intrathoracically infected *Ae. albopictus* mosquitoes.

Although the CHIKV E1-A226V mutation gives a selective advantage in *Ae. albopictus*, there was not a corresponding advantage in *Ae. aegypti.* The OID_50_ and midgut competition assay data indicate that E1-226V viruses were slightly less infectious for midgut cells of *Ae. aegypti* mosquitoes (Figures 1C, 1D, and 2C; Table 2). Additionally, in contrast to *Ae. albopictus*, E1-226V viruses do not have a detectable advantage for dissemination into salivary glands and heads of *Ae. aegypti*. In transmission competition experiments from Ae aegypti to suckling mice, E1-226V conferred a slight competitive advantage over E1-226A (Figure 5C). However, five out of six mice exposed to CHIKV infected Ae aegypti had equivalent amounts of both E1-226A and E1-226V viral RNAs. These results are markedly different compared to the results obtained in similar experiments using *Ae. albopictus* mosquitoes and further support the hypothesis that this E1-A226V was specifically selected as a result of adaptation of CHIKV to *Ae. albopictus* mosquitoes. To explain the small fitness advantage associated with the E1-A226V mutation which was observed in transmission experiments, we hypothesize that, similarly to *Ae. albopictus*, E1-226A and E1-226V viruses colonize different *Ae. aegypti* organs at different efficiencies. E1-226A appears to colonize midgut cells of Ae aegypti better than E1-226V viruses; however, following dissemination into salivary glands, the E1-226V virus gains an advantage for transmission to vertebrates.

The E1-A226V mutation was found to have a slightly negative effect on infectivity, a negligible effect on dissemination, but a slight positive effect on transmissibility of CHIKV by *Ae. aegypti* in the competition experiment. We suggest that these small (as compared with *Ae. albopictus*) differences associated with the E1-A226V mutation would not be sufficient to have a significant effect on the evolution of CHIKV transmitted by *Ae. aegypti* and would not result in accumulation of this mutation in the regions where *Ae. aegypti* serves as a primary vector for CHIKV. This may explain the lack of emergence of the E1-226V genotype in previous outbreaks and the predominance of E1-226A viruses during the 2006 CHIKV epidemic in India, in which *Ae. aegypti* is considered to be the main vector species [44]. Adaptation of African strains of CHIKV from forest dwelling mosquitoes species to *Ae. aegypti* has never been shown to be associated with any particular mutations, therefore we believe that the same negative impact of E1-A226V would be seen in African mosquito vectors which were responsible for transmission of CHIKV strains ancestral to Reunion isolates.

Our data does not exclude the possibility that the E1-A226V mutation might have a negative effect on the evolution of CHIKV transmitted by *Ae. aegypti*. Since our dissemination and transmission studies were performed using blood meal titers that were 1–2 Log_10_TCID_50_/ml higher than Log_10_OID_50_/ml values we suggest that the negative effect of decreased midgut infectivity of E1-A226V on virus transmissibility would be almost completely missed, simply because, under this condition, almost 100% of mosquitoes could become infected. In general, CHIKV requires significantly higher blood meal titers for infection of *Ae. aegypti* compared to *Ae. albopictus* [36,37] (Tables 1 and 2), which suggests that the slight decrease in midgut infectivity of E1-226V viruses would have a more profound effect on the evolution of CHIKV transmitted by *Ae. aegypti*, compared to the effect of a small advantage in the ability to compete with E1-226A viruses for transmission to suckling mice. Therefore, if the E1-A226V mutation occurred in CHIKV transmitted by *Ae. aegypti*, it would have a weak negative effect on viral fitness and would most likely not be preferentially selected. Additional experiments are required to evaluate this hypothesis.

Available data cannot exclude the possibility that E1-226A viruses may have an unknown beneficial effect on the fitness of CHIKV in vertebrate hosts over E1-226V viruses, and that the minor negative effect of E1-226A observed in transmission experiments by *Ae. aegypti* can be compensated for by more efficient viral replication in the vertebrate host, leading to an overall more efficient adaptation to the transmission cycle. However, comparison of the different effects of A or V residues at position E1–226 on CHIKV infectivity for, and transmission by *Ae. aegypti* and *Ae. albopictus* mosquitoes clearly suggests that polymorphisms at this position may determine the host range of the alphaviruses and may play an important role in adaptation of the viruses to a particular mosquito vector.

An interesting observation, which should be studied in more detail, was that adaptation of CHIKV to *Ae. albopictus* mosquitoes coincided with the acquisition of CHIKV dependence on cholesterol in the target membrane. It has been previously shown that various mutations in the same region of the E1 protein of SFV and Sindbis virus can modulate the cholesterol dependence of these viruses [33,45] and that SFV independence from cholesterol coincides with more rapid growth of the virus in *Ae. albopictus* [34]. Although there is an apparent association, it is currently unknown if cholesterol dependence of alphaviruses is directly responsible for modulation of fitness of alphaviruses in mosquito vectors. A possible explanation for the opposite effects of the cholesterol-dependent phenotype of SFV and CHIKV on fitness in *Ae. albopictus* may reflect the use of different techniques for mosquito infection. In our study, mosquitoes were orally infected via cholesterol rich blood meals, whereas in the previous study SFV was intrathoracically inoculated into the mosquito [34]. It is also possible that cholesterol-dependent and -independent viruses would replicate differently in different mosquito organs. As such, our data indicate that more efficient colonization of *Ae. albopictus* midgut cells by cholesterol-dependent LR-ApaI-226V is followed by relatively more rapid growth of cholesterol-independent LR −226A virus in mosquito bodies between 3 and 5 dpi (Figure 4B). Three to 5 dpi coincides with virus escape from the mosquito midgut.

Alignment of amino acid sequences that constitute the ij loop of E1 protein from different members of the alphaviruses genus revealed that position E1–226 is not conserved ([33] and data not shown) and can vary even between different strains of the same virus. In this regard, it would be reasonable to determine the cholesterol requirement of other clinically important alphaviruses, especially Venezuelan equine encephalitis virus (VEEV) and eastern equine encephalitis virus (EEEV), which show significant intra-strain variation at position E1–226 among natural isolates of these viruses, and determine mutations which can modulate their cholesterol dependence. In recent studies by Kolokoltsov et al. [46], it was suggested that VEEV, a New world alphavirus, might be cholesterol independent, although the use of Vero cells instead of C6/36 cells, and the use of different protocols for cell membrane cholesterol depletion, make it difficult to compare the results of this study with our findings. Also it would be of interest to determine possible relationships between mutations which modulate cholesterol dependence of alphaviruses other than CHIKV and on their infectivity for *Ae. aegypti* and *Ae. albopictus* mosquitoes and perhaps other epidemiologically important mosquito vectors*.*

The molecular mechanisms responsible for the association between host range and cholesterol dependence of CHIKV are unknown [47]. It has been proposed that upon exposure to low pH, the E1 protein of cholesterol-dependent viruses senses the target membrane lipid composition and goes through a cholesterol-dependent priming recognition reaction [48] which is not required for cholesterol-independent viruses. It is possible that CHIKV infects *Ae. aegypti* and *Ae. albopictus* midgut cells using different endocytic pathways, which targets virus to cellular compartments with different lipid contents in which fusion occurs. Specific lipids such as cholesterol may differentially affect fusion of cholesterol-dependent and cholesterol-independent CHIKV strains in these compartments and therefore define the outcome of infection. Although our observations are suggestive, more comprehensive studies should be completed to determine the exact molecular mechanisms responsible for penetration of E1-226A and E1-226V viruses into *Ae. aegypti* and *Ae. albopictus* cells.

Although previous laboratory studies have demonstrated susceptibility of *Ae. albopictus* to CHIKV infection [36,37], our data demonstrate that the E1-A226V mutation promoted infection and accelerated dissemination of CHIKV in *Ae. albopictus* mosquitoes and conferred a selective advantage over infection of *Ae. aegypti.* Whilst the mutation did not increase the maximum viral titer attainable in the mosquitoes, the synergistic effects of increased infectivity and faster dissemination of the E1-A226V virus in *Ae. albopictus* would accelerate virus transmission to a naïve human population which would have contributed to initiating and sustaining the 2005–2006 CHIKV epidemic on Reunion island. That a single amino acid change can act through multiple phenotypic effects to create an epidemic situation has implications for other arthropod-transmitted viruses and the evolution of human infectious diseases [49].

## Methods

### Viruses and plasmids.

The viruses and plasmids encoding full-length infectious clones of the LR2006 OPY1 strain CHIK-LR ic (GenBank accession number EU224268; http://www.ncbi.nlm.nih.gov/Genbank/index.html) and GFP-expressing full-length clone LR-GFP-226V (CHIK-LR 5′GFP, GenBank accession number EU224269) have been previously described [15,35]. The plasmids 37997–226A (pCHIK-37997ic, GenBank accession number EU224270) encoding full-length infectious clones of the West African strain of CHIKV 37997 and a GFP-expressing full-length clone 37997-GFP-226A (pCHIK-37997–5GFP, GenBank accession number EU224271) were derived from previously described plasmids pCHIKic and 5′CHIK EGFP [35] by introducing CHIKV encoding cDNA into a modified pSinRep5 (Invitrogen) at positions 8055–9930. Viruses derived from 37997–226A and 37997-GFP-226A are identical to viruses derived form pCHIKic and 5′CHIK EGFP. To facilitate rapid screening of viruses in mosquitoes, the gene encoding enhance green fluorescent protein (eGFP), that is known not to compromise CHIKV phenotype in mosquitoes [15], was incorporated into clones as previously described [15]. Plasmids were constructed and propagated using conventional PCR-based cloning methods [50]. The entire PCR-generated regions of all constructs were verified by sequence analysis. The maps, sequences and detailed description of the clones are available from the authors upon request. For studies comparing the relative fitness of the mutant (E1-226V) virus and the pre-epidemic genotype (E1-226A), a silent mutation (6454C) was introduced into the CHIK-LR ic, to add an *Apa*I restriction site into the coding sequence of CHIK-LR ic. The resultant plasmid was designated LR-ApaI-226V. The E1-V226A mutation was introduced into CHIK-LR ic and LR-GFP-226V to generate plasmids designated as LR-226A and LR-GFP-226A, respectively. The mutation E1-A226V was also introduced into plasmids 37997–226A and 37997-GFP-226A. The resulted plasmids were designated 37997–226V and 37997-GFP-226V.

All plasmids were purified by centrifugation in CsCl gradients, linearized with *Not*I and *in vitro* transcribed from the minimal SP6 promoter using the mMESSAGE mMACHINE kit (Ambion) following the manufacturer's instructions. The yield and integrity of synthesized RNA were analyzed by agarose gel electrophoresis in the presence of 0.25 μg/ml of ethidium bromide. RNA (10 μg) was transfected into 1x10^7^ BHK-21 cells by electroporation as previously described [15]. Cells were transferred to 25 cm^2^ tissue culture flasks with 10 ml of Leibovitz L-15 (L-15) medium, and supernatants were collected at 24 and 48 h post-electroporation and stored at −80 °C. In parallel, 1x10^5^ electroporated BHK-21 cells were serially 10-fold diluted and seeded in six-well plates for infectious centers assay as previously described [15].

### Cells and mosquitoes.

BHK-21 (baby hamster kidney) cells were maintained at 37 °C in L-15 medium supplemented with 10% fetal bovine serum (FBS), 100 U penicillin, and 100 μg/ml streptomycin. C6/36 cells (*Ae. albopictus*) were grown in the same medium at 28 °C. *Ae. aegypti* (white-eyed Higgs variant of the Rexville D strain) and *Ae. albopictus* (Galveston strain) were reared at 27 °C and 80% relative humidity under a 16h light: 8h dark photoperiod, as previously described [35]. Adults were kept in paper cartons supplied with 10% sucrose on cotton balls. To promote egg production females were fed on anaesthetized hamsters once per week.

Rexville D strain of *Ae. aegypti* mosquitoes were originally selected for susceptibility to flavivirus infection [51]. Since there are no known consequences of this original selection with respect to susceptibility to CHIKV, a white eyed variant of the strain that facilitates detection of GFP was used in our experiments.

### In vitro virus growth of CHIKV in standard and cholesterol-depleted C6/36 cells.

To investigate if the mutation influenced cholesterol dependence of the virus, cholesterol-depleted C6/36 cells were prepared by five passages in L-15 medium containing 10% FBS treated with 2% CAB-O-Sil (Acros Organics) for 12 h at room temperature as previously described [52]. CHIKV growth curves were determined by infecting cholesterol-depleted and normal C6/36 cells at a multiplicity of infection (MOI) of 0.1 and 1.0, respectively, by rocking for 1 h at 25 °C. The cells were washed three times with L-15 medium and 5.5 ml of fresh L-15 supplied with 10% of standard or CAB-O-Sil treated FBS was added to the flask. At the indicated times post-infection, 0.5 ml of medium was removed and stored at −80 °C until titrated. The volume of medium was then restored by adding 0.5 ml of appropriate medium.

### Titrations.

Viral titers from mosquito samples and from tissue culture supernatant were determined using Vero cells and expressed as tissue culture infectious dose 50 percent endpoint titers (Log_10_TCID_50_) as previously described [53]. Additionally, for viral competition experiments, titers of LR-Apa-226V LR-226A viruses were determined using standard plaque assay on Vero cells as previously described [54].

### Oral infection of mosquitoes.

*Ae. aegypti* and *Ae. albopictus* were infected in an Arthropod Containment Level 3 insectary as described previously [35,55]. To make infectious blood meals for the viruses lacking eGFP, viral stocks derived from electroporated BHK-21 cells were mixed with an equal volume of defibrinated sheep blood and supplemented with 3 mM ATP as a phago-stimulant. To produce infectious blood meals for the eGFP-expressing viruses, the viruses were additionally passed on BHK-21 cells. The cells were infected at a MOI ≈ 1.0 with virus derived from electroporation. At 2 dpi, cell culture supernatants were mixed with an equal volume of defibrinated sheep blood and presented to 4- to 5-day-old female mosquitoes that had been starved for 24 h, using a Hemotek membrane feeding system (Discovery Workshops) and hamster skin membrane. Mosquitoes were allowed to feed for 45 min, and engorged mosquitoes (stage ≥3+ [56]) were sorted and returned to a cage for maintenance. Blood meals and three to four mosquitoes were immediately removed for titration and/or RNA extraction. Depending on the purpose of the experiments, mosquitoes were collected at different days post-infection and either titrated to determine viral titer, dissected for analysis of eGFP expression in the midguts or salivary glands [15], or used for RNA extraction in competition experiments.

To estimate the Oral Infectious Dose 50% values (OID_50_), serial 10-fold dilutions of viruses were made in L-15 medium followed by mixing the samples with defibrinated sheep blood. Mosquitoes were dissected at 7 dpi and eGFP expression in infected midguts was analyzed by fluorescence microscopy. A mosquito was considered infected if at least one foci of eGFP-expressing cells was present in the midgut. The experiments were performed twice for each virus. OID_50_ values and confidence intervals were calculated using PriProbit (version 1.63).

### Viral competition experiments.

To test the hypothesis that the E1-A226V mutation might be associated with a competitive advantage in mosquito vectors, competition assays were designed similar to those described previously in mice [57], with minor modifications (Figure 2A). Both *Ae. aegypti* and *Ae. albopictus* mosquitoes were presented with a blood meal containing 10^7^ plaque-forming units (pfu)/ml of LR-Apa-226V and 10^7^ pfu/ml of LR-226A viruses. It had been previously found that for these two viruses the ratio of viral RNAs corresponds to the ratio of viral titers (data not shown). Midguts were collected at 7 dpi and analyzed in pools of eight to ten, and heads were collected at 12 dpi and analyzed in pools of five. RNA was extracted from the tissue pools using TRIzol reagent (Invitrogen) followed by additional purification using a Viral RNA mini kit (QIAGEN). RNAs from blood meal samples were extracted using Viral RNA Mini Kit followed by treatment with DNAse (Ambion) to destroy any residual plasmid DNA contaminant in the viral samples. RNA was reversed transcribed from random hexamer primers using Superscript III (Invitrogen) according to the manufacturer's instructions. cDNA was amplified from 41855ns-F5 (5′- ATATCTAGACATGGTGGAC) and 41855ns-R1 (5′-TATCAAAGGAGGCTATGTC) primers using Taq DNA polymerase (New England Biolabs). PCR products were purified using Zymo clean columns (Zymo Research) and were quantified by spectrophotometry. Equal amount of PCR products were digested with *Apa*I, separated in 2% agarose gels that were stained using ethidium bromide. Thus the LR-Apa-226V and LR-226A viruses could be distinguished by size on an agarose gel (Figure 2A). Gel images were analyzed using TolaLab (version 2.01). Relative fitness of LR-Apa-226V and LR-226A viruses was calculated as a ratio between 226V and 226A bands in the sample, divided by the control ratio of 226V and 226A in the blood meal.

### Virus competition in an animal transmission model.

*Ae. aegypti* and *Ae. albopictus* mosquitoes were presented with a blood meal containing 10^7^ pfu/ml of LR-Apa-226V and 10^7^ pfu/ml of LR-226A viruses. At 13 dpi, ten to 15 mosquitoes were placed in separate paper cartons and starved for 24 h. The next day the mosquitoes in each carton were presented with individual 2- to 3-day-old suckling mouse (Swiss Webster). Feeding continued until 2–3 mosquitoes per carton were fully engorged (stage ≥3+[56]). In a parallel experiment six 2- to 3-day-old suckling mice were subcutaneously infected with 20 μl of mixture containing ≈ 25 pfu of LR-Apa-226V and ≈ 25 pfu of LR-226A viruses. Mice were returned to their cage and sacrificed on day 3 post-exposure. Blood from each individual mouse (≈ 50 μl) was collected and immediately mixed with 450 μl of TRIzol reagent for RNA extraction. The RNA was processed as described above. All animal manipulations were conducted in accordance with federal laws, regulations, and in compliance with National Institutes of Health and University of Texas Medical Branch Institutional Animal Care and Use Committee guidelines and with the Association for Assessment and Accreditation of Laboratory Animal Care standards.

## Supporting Information

Figure S1 Schematic Representation of the Viruses Used in This Study (917 KB PDF)

Figure S2 Growth of the eGFP-Expressing Viruses in BHK-21(A, C) and C6/36 (B, D) Cells Confluent monolayers of BHK-21 and C6/36 cells in T25 tissue culture flacks were infected with LR-GFP-226V and LR-GFP-226A (A, B) or 37997-GFP-226A and 37997-GFP-226V viruses derived from electroporation at a MOI of 0.1. At the indicated times post-infection, 0.5 ml of medium was removed and stored at −80 °C for later titration on Vero cells. Viral titers are expressed as Log_10_TCID_50_/ml.
(372 KB PDF)

Figure S3 Growth of the CHIK-LR ic, LR-ApaI-226V and LR-226A Viruses in BHK-21(A) and C6/36 (B) Cells Confluent monolayers of BHK-21 and C6/36 cells in T25 tissue culture flacks were infected with LR-GFP-226V and LR-GFP-226A (A, B) or 37997-GFP-226A and 37997-GFP-226V viruses derived from electroporation at a MOI of 1.0. At the indicated times post-infection, 0.5 ml of medium was removed and stored at −80 °C until titrated on Vero cells. Viral titers are expressed as Log_10_TCID_50_/ml ± standard deviation of three independent experiments.
hpi - hours post-infection.
(177 KB PDF)

Figure S4 Competition between CHIK-LR ic and LR-ApaI-226V for Growth in BHK-21 and C6/36 Cells Cells were infected with a 1:1 mixture of both viruses at a MOI of 0.001. 2 dpi, cell culture supernatant was collected and samples proceeded as described. The experiment was repeated three times for each of the cell types.
inoc - initial ratio of CHIK-LR ic and LR-ApaI-226V in the inoculum used for infection of cells.
Relative fitness (RF) of CHIK-LR ic and LR-ApaI-226V was calculated as an average ratio between CHIK-LR ic and LR-ApaI-226V bands in the supernatant obtained from BHK-21 cells (RF_1_) and C6/36 cells (RF_2_), divided by the control ratio between CHIK-LR ic and LR-ApaI-226V in the inoculum.
(3.6 MB PDF)

Table S1 Specific Infectivity and Virus Titers after Electroporation a - amino acids at position of E1–226.
b - Specific infectivity of *in vitro* transcribed RNA. 10^7^ BHK-21 cells were transfected with 10 μg of RNA. Electroporated BHK-21 cells were 10-fold serially diluted, seeded in 6-well tissue culture plates containing 5x10^5^ naive BHK-21 cells per well and covered with 0.5% agarose in L-15. Plaques were scored on day 2 post-transfection.
c - Supernatants of electroporated BHK-21 cells were collected on days 1 and 2. Virus titers were determined by titration on Vero cells and expressed as Log_10_TCID_50_/ml.
hpi - hours post-infection.
(34 KB DOC)

Table S2 Infection Rates and Average Titers of CHIKV-LR ic or LR-ApaI-226V in Orally Infected *Ae. aegypti* and *Ae. albopictus*
*Ae. aegypti* mosquitoes were orally presented with 7.24±0.4 Log_10_TCID_50_/ml of CHIKV-LR ic (summary of two experiments) and 6.52 Log_10_TCID_50_/ml of LR-ApaI-226V.
*Ae. albopictus* mosquitoes were orally presented with 7.24±0.4 Log_10_TCID_50_/ml of CHIKV-LR ic (summary of two experiments) and 7.52 Log_10_TCID_50_/ml LR-ApaI-226V.
At 7 and 14 dpi, mosquitoes were collected and triturated in 1mL of L-15 medium for titration on Vero cells.
Titers are reported as Log_10_TCID_50_/ml ± standard deviation.
(31 KB DOC)
