# Supplementary material for: A Single Mutation in Chikungunya Virus Affects Vector Specificity and Epidemic Potential
Source: PLoS Pathog. 2007 Dec 7;3(12):e201. doi: 10.1371/journal.ppat.0030201 (PMC2134949; doi:10.1371/journal.ppat.0030201)
Supplement: Supplementary file 1 [file ppat.0030201.sg001.pdf]

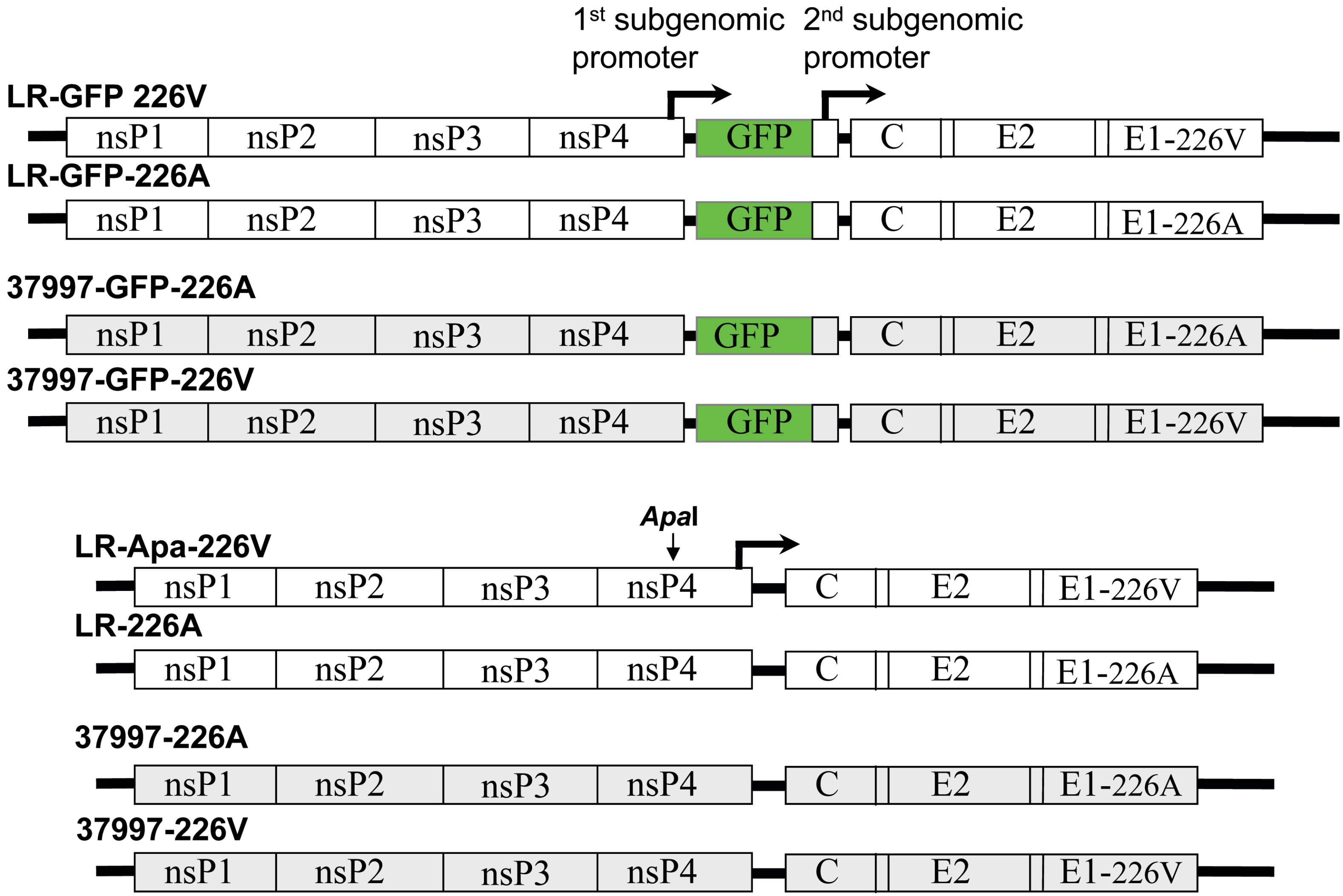
1st subgenomic promoter
2nd subgenomic promoter
LR-GFP 226V
nsP1 nsP2 nsP3 nsP4 GFP C E2 E1-226V
LR-GFP-226A
nsP1 nsP2 nsP3 nsP4 GFP C E2 E1-226A
37997-GFP-226A
nsP1 nsP2 nsP3 nsP4 GFP C E2 E1-226A
37997-GFP-226V
nsP1 nsP2 nsP3 nsP4 GFP C E2 E1-226V
ApaI
LR-Apa-226V
nsP1 nsP2 nsP3 nsP4 C E2 E1-226V
LR-226A
nsP1 nsP2 nsP3 nsP4 C E2 E1-226A
37997-226A
nsP1 nsP2 nsP3 nsP4 C E2 E1-226A
37997-226V
nsP1 nsP2 nsP3 nsP4 C E2 E1-226V
