# Supplementary material for: A Single Mutation in Chikungunya Virus Affects Vector Specificity and Epidemic Potential
Source: PLoS Pathog. 2007 Dec 7;3(12):e201. doi: 10.1371/journal.ppat.0030201 (PMC2134949; doi:10.1371/journal.ppat.0030201)
Supplement: Supplementary file 2 [file ppat.0030201.sg002.pdf]

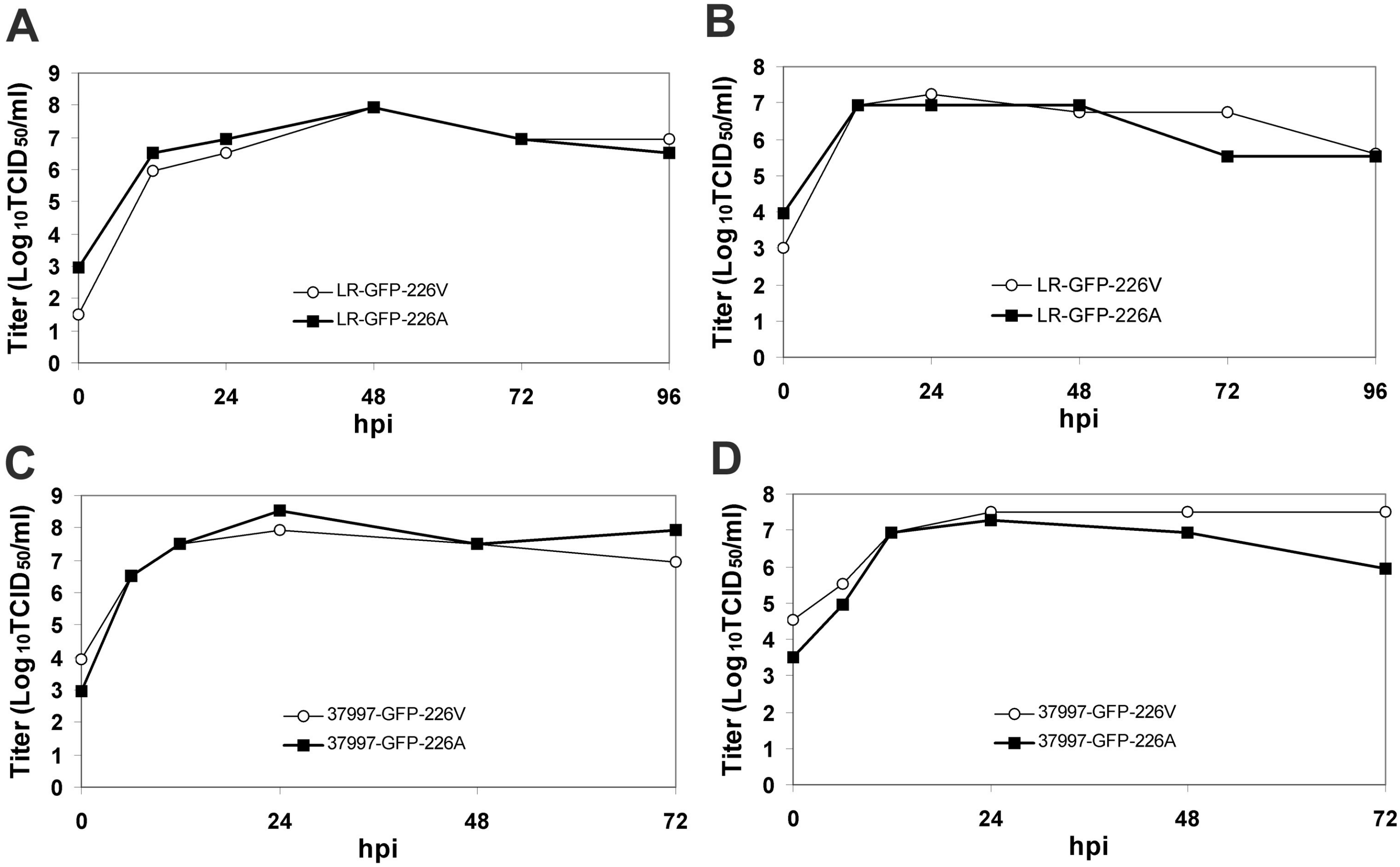
A
B
C
D
Titer (Log10TCID50/ml)
hpi
LR-GFP-226V
LR-GFP-226A
37997-GFP-226V
37997-GFP-226A
0
24
48
72
96
