# Supplementary material for: A Single Mutation in Chikungunya Virus Affects Vector Specificity and Epidemic Potential
Source: PLoS Pathog. 2007 Dec 7;3(12):e201. doi: 10.1371/journal.ppat.0030201 (PMC2134949; doi:10.1371/journal.ppat.0030201)
Supplement: Supplementary file 3 [file ppat.0030201.sg003.pdf]

A

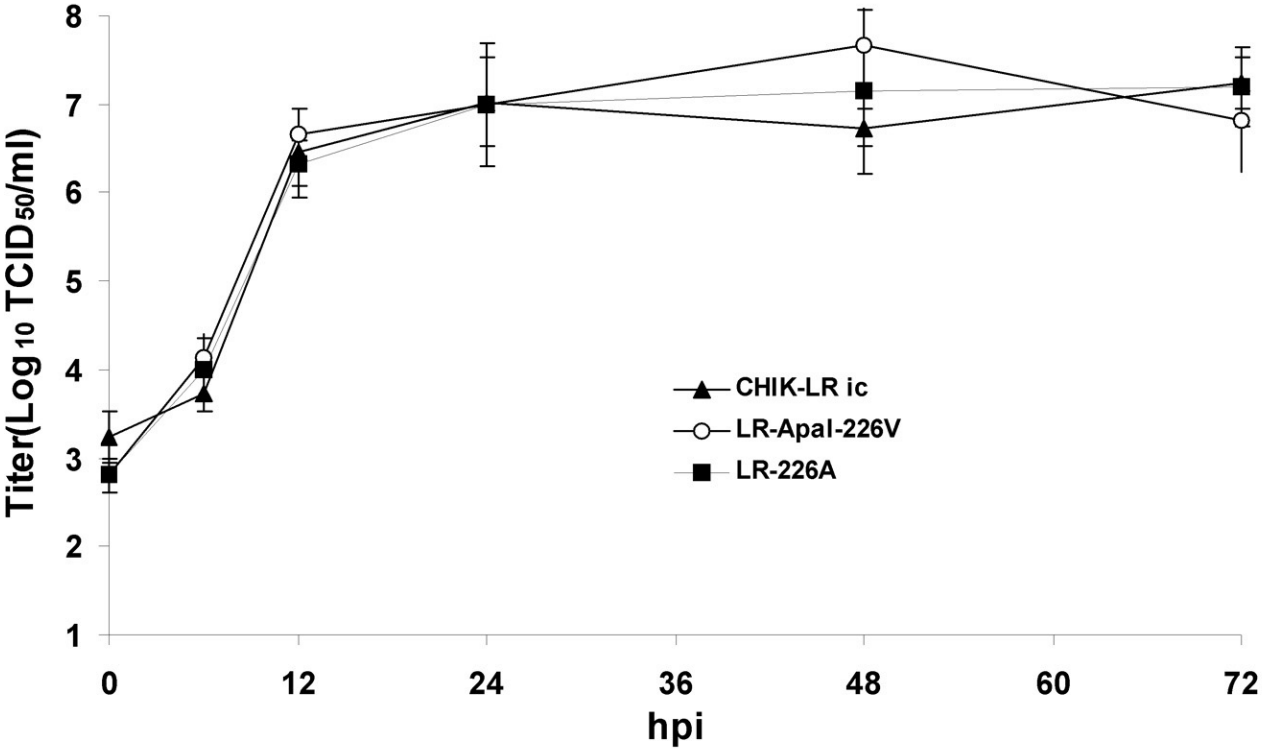
8
7
6
5
4
3
2
1
Titer(Log10 TCID50/ml)
0
12
24
36
48
60
72
hpi
CHIK-LR ic
LR-ApaI-226V
LR-226A

B

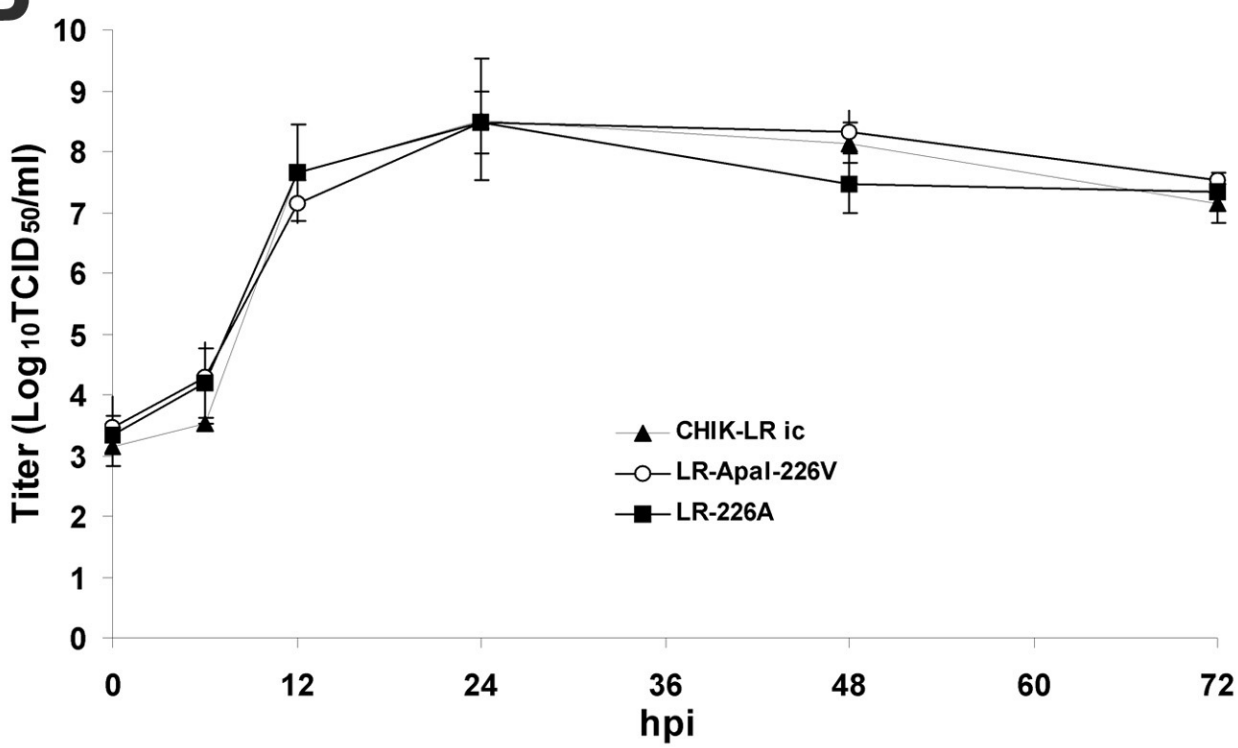
10
9
8
7
6
5
4
3
2
1
0
Titer (Log10TCID50/ml)
0
12
24
36
48
60
72
hpi
CHIK-LR ic
LR-ApaI-226V
LR-226A
