# Supplementary material for: A Single Mutation in Chikungunya Virus Affects Vector Specificity and Epidemic Potential
Source: PLoS Pathog. 2007 Dec 7;3(12):e201. doi: 10.1371/journal.ppat.0030201 (PMC2134949; doi:10.1371/journal.ppat.0030201)
Supplement: Supplementary file 4 [file ppat.0030201.sg004.pdf]

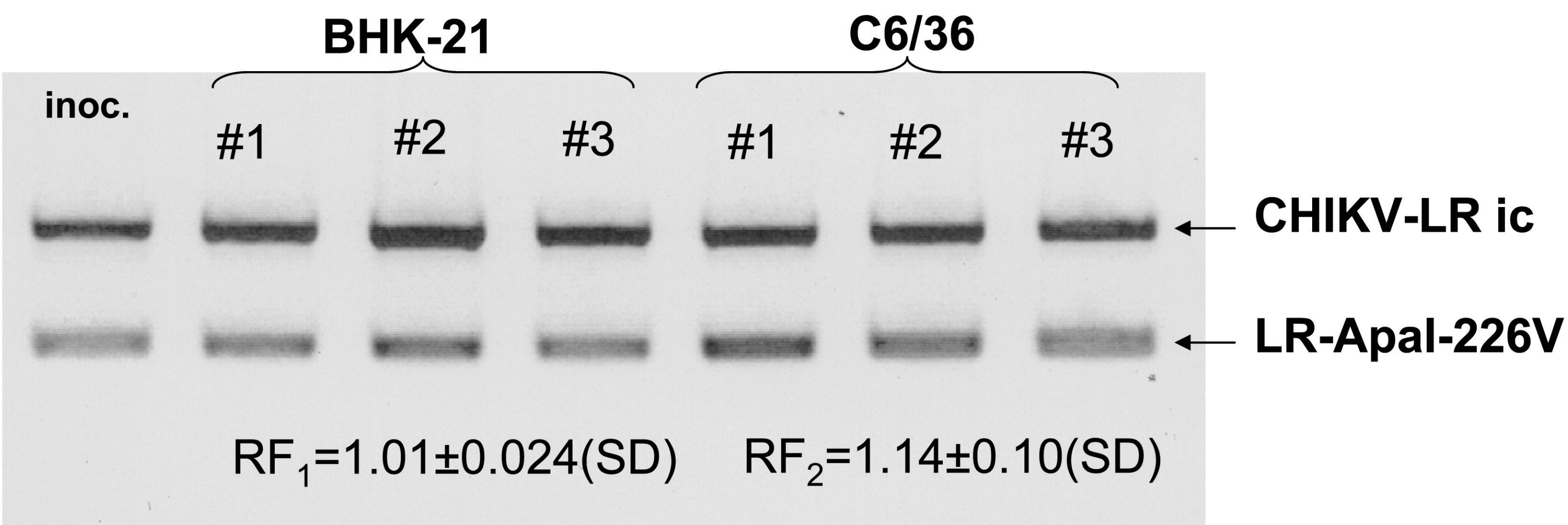
BHK-21
C6/36
inoc.
#1
#2
#3
#1
#2
#3
CHIKV-LR ic
LR-ApaI-226V
RF1=1.01±0.024(SD)
RF2=1.14±0.10(SD)
