# Supplementary material for: A Single Mutation in Chikungunya Virus Affects Vector Specificity and Epidemic Potential
Source: PLoS Pathog. 2007 Dec 7;3(12):e201. doi: 10.1371/journal.ppat.0030201 (PMC2134949; doi:10.1371/journal.ppat.0030201)
Supplement: Supplementary file 5 [file ppat.0030201.st001.doc]

**Table S1.** Specific infectivity and virus titers after electroporation.

| Template used for *in vitro* transcription | E1-226 | Specific infectivity  (pfu/μg of RNA ) | Virus titer  24h 48h | |
| --- | --- | --- | --- | --- |
| LR-ApaI-226V | V | 9.1 x 10^5^ | 6.95 | 7.52 |
| LR-226A | A | 8.3 x 10^5^ | 6.52 | 7.52 |
| CHIK-LR ic | V | 8.0 x10^5^ | 6.95 | 7.95 |
| LR-GFP-226V | V | 4.8 x 10^5^ | 6,52 | 6.52 |
| LR-GFP-226A | A | 3.3 x 10^5^ | 6.95 | 6.95 |
| 37997-226A | A | 5.0 x 10^5^ | 7.52 | 7.52 |
| 37997-226V | V | 9.0 x 10^5^ | 7.95 | 7.95 |
| 37997-GFP-226A | A | 5.0 x 10^5^ | 7.52 | 7.52 |
| 37997-GFP-226V | V | 5.0 x 10^5^ | 6.95 | 7.52 |
