# Supplementary material for: A Single Mutation in Chikungunya Virus Affects Vector Specificity and Epidemic Potential
Source: PLoS Pathog. 2007 Dec 7;3(12):e201. doi: 10.1371/journal.ppat.0030201 (PMC2134949; doi:10.1371/journal.ppat.0030201)
Supplement: Supplementary file 6 [file ppat.0030201.st002.doc]

**Table S2**. Infection rates and average titers of CHIKV-LR ic or LR-ApaI-226V in orally infected *Ae. aegypti* and *Ae. albopictus.*

| Mosquitoes | Day p.i. | CHIK-LR ic | | LR-ApaI-226V | |
| --- | --- | --- | --- | --- | --- |
|  |  | Infected / Total (%) | Titer±S.D | Infected / Total (%) | Titer±S.D |
| *Ae. aegypti* | 7 | 14/18 (78) | 6.13±0.78 | 7/10 (70) | 5.90±0.43 |
|  | 14 | 6/10 (60) | 5.64±0.36 | 7/10 (70) | 4.90±0.64 |
|  | | | | | |
| *Ae. albopictus* | 7 | 15/16 (94) | 5.08±0,78 | 10/10 (100) | 4.91±0.31 |
|  | 14 | 15/16 (94) | 4.35±0,58 | 10/10 (100) | 4.36±0.37 |
